# Postoperative delirium in elderly orthopedic patients: a narrative review of prevention and multidisciplinary nursing interventions

**DOI:** 10.3389/fpsyt.2026.1817807

**Published:** 2026-06-30

**Authors:** Chen Wang, Chuanqiang Dai, Guifang Wu, Youshu Zhang, Yao Zhang, Yao Dong

**Affiliations:** 1 Department of Surgery, Ziyang Central Hospital, Ziyang, Sichuan, China; 2 Department of Orthopedics, Ziyang Central Hospital, Ziyang, Sichuan, China

**Keywords:** clinical translation, elderly patients, implementation science, non-pharmacological strategies, nursing interventions, orthopedic surgery, postoperative delirium, risk prediction

## Abstract

**Background:**

Postoperative delirium (POD) affects 20–60% of elderly orthopedic surgery patients, yet most cases go unrecognized—over 70% in some reports. This gap between what we know and what we do in daily practice is frustrating, but also hopeful: because POD is largely preventable. Early identification and evidence-based prevention remain urgent priorities.

**Objective:**

This review brings together recent insights into why POD happens and what bedside nurses and surgical teams can actually do about it. We focus on actionable, non-pharmacological strategies that work in real-world settings.

**Methods:**

We searched PubMed and Google Scholar for peer-reviewed studies published between 2022 and 2025, focusing on POD mechanisms, risk prediction, and non-drug interventions. Given the heterogeneity of study designs and outcomes, we used a narrative synthesis approach rather than formal meta-analysis. Twenty high-quality articles were selected for critical appraisal.

**Key Findings:**

POD arises from a tangled web of causes, with neuroinflammation and neurotransmitter imbalance at its core. Newer risk tools can now flag high-risk patients before surgery. The eCASH bundle—Early mobilization, Cognitive stimulation, Adequate sleep, Social support, and Homelike environment—consistently cuts POD rates. Multidisciplinary teamwork works far better than any single discipline going it alone. And while implementation barriers like understaffing and knowledge gaps are real, structured training and simple protocols can overcome them.

**Conclusion:**

A three-part strategy—risk-stratified screening, eCASH bundled interventions, and multidisciplinary collaboration—offers the best shot at reducing POD in elderly orthopedic patients. Looking ahead, biomarkers, remote monitoring, and implementation science will take us further.

## Highlights

**What We Know:**
- POD occurs in 20–60% of elderly orthopedic patients but goes undiagnosed in >70% of cases
- Neuroinflammation and neurotransmitter dysregulation are core mechanisms
- Risk stratification tools can identify high-risk patients before surgery
- Non-drug interventions consistently prevent POD

**What This Review Adds:**
- A practical synthesis of mechanistic insights and clinical translation pathways
- An implementation framework for multidisciplinary eCASH-based prevention
- Evidence-based solutions for real-world barriers (staffing, knowledge gaps)
- Health economic justification for investing in prevention

**Clinical Application:**
- Screen all post-op patients ≥65 years systematically, not just when concerns arise
- For high-risk patients, activate early, intensive eCASH bundle with multidisciplinary coordination
- Use standardized tools (Nu-DESC, CAM) at scheduled intervals with clear escalation pathways
- Join local quality improvement efforts to adapt these strategies to your patient population and resources

## 1 Introduction

### 1.1 The clinical paradox of postoperative delirium

Here’s a strange contradiction in modern surgery: a patient can sail through a complex hip replacement, yet days later, they’re confused, agitated, or strangely withdrawn—sometimes without anyone noticing. Postoperative delirium (POD)—that sudden, fluctuating state of confusion, inattention, and disorganized thinking—remains one of medicine’s most overlooked complications.

The numbers are hard to ignore. Among older adults having orthopedic surgery, especially emergency hip fracture repair or joint replacement, POD rates run from 20% to 60% (1, 2). But here’s the sobering part: a multi-center survey found that more than 70% of POD cases in surgical patients never got diagnosed (3). That’s not just an occasional slip—it’s a systemic blind spot.

Why should we care? Because POD isn’t a benign episode that passes without consequence. Patients who develop delirium stay in the hospital longer, cost more to treat, recover function more slowly, and face higher risks of long-term cognitive decline (4). As our population ages and more older adults undergo surgery, POD has stopped being a curious footnote and become a public health priority—one we can actually do something about.

### 1.2 Purpose and scope of this review

This review has three goals. First, to explain—clearly and concretely—what’s happening in the aging brain that makes it so vulnerable to POD. Second, to walk through practical risk tools that help identify which patients need the most attention. And third, to translate the latest evidence into bedside nursing interventions that work in real hospitals, not just research trials. We wrote this for surgical teams who want a framework that’s both scientifically sound and doable on a Tuesday morning.

## 2 The diagnostic challenge: toward enhanced recognition

Before we can prevent POD, we have to recognize it. Yet unlike a heart attack or sepsis, delirium has no single definitive lab test. Diagnosis depends on careful observation—exactly the thing that gets squeezed out when nurses are juggling six patients and a crashing blood pressure.

### 2.1 Heterogeneous clinical presentation

The most widely used tools are the Confusion Assessment Method (CAM) and CAM-ICU, which track four features: acute onset, inattention, disorganized thinking, and altered consciousness (5, 6).In ICUs and high-acuity units, the Nu-DESC scale and MMSE offer additional options—but they all require time and training, both in short supply on understaffed night shifts. For the Nu-DESC, a cutoff of ≥2 suggests possible delirium (7).

There’s also a subtle cognitive trap at work. Many clinicians see a quiet, sleepy patient and chalk it up to normal postoperative sedation. But that’s often hypoactive delirium, the most common form—and the one least likely to raise alarms (5). Hyperactive delirium, on the other hand, gets immediate attention but is often dismissed as “anxiety” or “being difficult” rather than recognized for what it is: acute brain dysfunction.

### 2.2 Diagnostic gap and clinical implications

One study drove the point home: in resource-limited hospitals, the real POD rate was two to three times higher than what got recorded (3). That’s a staggering miss. If we don’t see it, we can’t treat it.

What this means for practice: Routine, scheduled screening using validated tools—not just when someone “looks off”—is the absolute minimum foundation for any serious POD prevention program.

## 3 Neurobiological mechanisms: from molecular mayhem to bedside clues

Why are older orthopedic patients so vulnerable? The answer lives at multiple biological levels, and recent research points to a few key pathways that work together in a vicious cycle (8).

### 3.1 Neuroinflammation as the central driver

The neuroinflammatory hypothesis has the strongest evidence behind it. Surgery triggers a flood of inflammatory signals—IL-6, IL-1β, TNF-α—into the bloodstream (2, 9). In a younger person, the blood-brain barrier and waste-clearance systems keep those signals out of the brain. But aging degrades both the barrier and the glymphatic system that clears debris (2, 9). So those inflammatory molecules slip through, activate microglia (the brain’s immune cells), and set off a cascade that directly impairs attention, executive function, and consciousness. Studies have found higher levels of these inflammatory markers in the cerebrospinal fluid of delirious patients (9), and animal work shows that blocking microglial activation prevents delirium-like behavior in old—but not young—animals.

### 3.2 Neurotransmitter system dysregulation

The brain’s chemical balance also goes haywire. Surgical stress, pain, sleep loss, and medications all disrupt the cholinergic, dopaminergic, noradrenergic, and serotonergic systems that maintain attention and clear thinking (9). A practical takeaway: anticholinergic drugs (often given for postoperative nausea) can push an already vulnerable brain into delirium by further crippling acetylcholine pathways. This is one place where medication choices really matter.

### 3.3 Protein homeostasis and cellular stress responses

Aging makes the brain’s protein quality control system brittle. Under the stress of surgery and inflammation, cells can’t keep up with misfolded proteins. They aggregate, trigger endoplasmic reticulum stress, and overwhelm the system. This explains why patients with baseline cognitive impairment—whose protein management systems are already strained—are at such high risk.

### 3.4 An integrated vicious cycle

None of these mechanisms operates in isolation. They feed each other: surgical stress → systemic inflammation → blood-brain barrier breakdown + neuroinflammation → neurotransmitter chaos → sleep-wake disruption → even more neuroinflammation and cognitive trouble. Once the cycle starts, it can keep spinning unless someone actively breaks it. That’s why multi-pronged prevention strategies beat single-target approaches every time.

## 4 Risk stratification: getting smarter about who needs what

Not every older patient faces the same delirium risk. Sorting patients into low, moderate, and high risk lets us put our limited resources where they’ll do the most good.

### 4.1 Patient-level and clinical risk factors

Age shows a clear dose-response relationship. An 85-year-old is at much higher risk than a 65-year-old—but age is really a proxy for accumulated physiological wear: less cognitive reserve, worse inflammatory regulation, slower waste clearance (2).

Pre-existing cognitive impairment may be the single strongest modifiable risk factor. Even subtle decline—the kind that doesn’t reach dementia but is noticeable to family—dramatically raises risk, presumably because cognitive reserve is already depleted. Recent nomogram-based models that combine nutritional status and cognitive screening have achieved clinically useful accuracy (2).

Medical comorbidities also matter: prior stroke, diabetes, and chronic kidney disease all independently increase POD risk, likely through impaired homeostasis and chronic low-grade neuroinflammation.

### 4.2 Perioperative factors

Longer surgeries, with more tissue trauma and anesthetic exposure, correlate with higher POD rates. Anesthetic technique makes a difference: regional (neuraxial) anesthesia appears safer than general, probably because it avoids systemic agents and preserves conscious processing (9).

Postoperative pain intensity, sleep deprivation, and social isolation (especially when families can’t visit) are modifiable triggers that amplify underlying vulnerability.

### 4.3 Pharmacological considerations for POD prevention

A few medication principles deserve explicit mention. Dexmedetomidine—a sedative with potent anti-inflammatory properties—has been associated with lower POD rates in multiple studies (9, 10). It’s not a magic bullet, but it’s a valuable tool, especially in ICUs. Benzodiazepines, by contrast, should be avoided in high-risk patients; they’re linked to worse delirium outcomes. Opioid-sparing anesthesia and postoperative analgesic regimens help reduce the risk of hypoxia and hypercapnia (which raise intracranial pressure). Ketamine also carries higher risk in vulnerable patients and is best avoided unless clearly indicated. Finally, low hematocrit and electrolyte disturbances—especially sodium and potassium imbalances—should be corrected promptly in any patient showing signs of POD. These aren’t exciting interventions, but they’re solid, evidence-based, and entirely within a surgical team’s reach (9).

### 4.4 Clinical application

**Figure 1:**
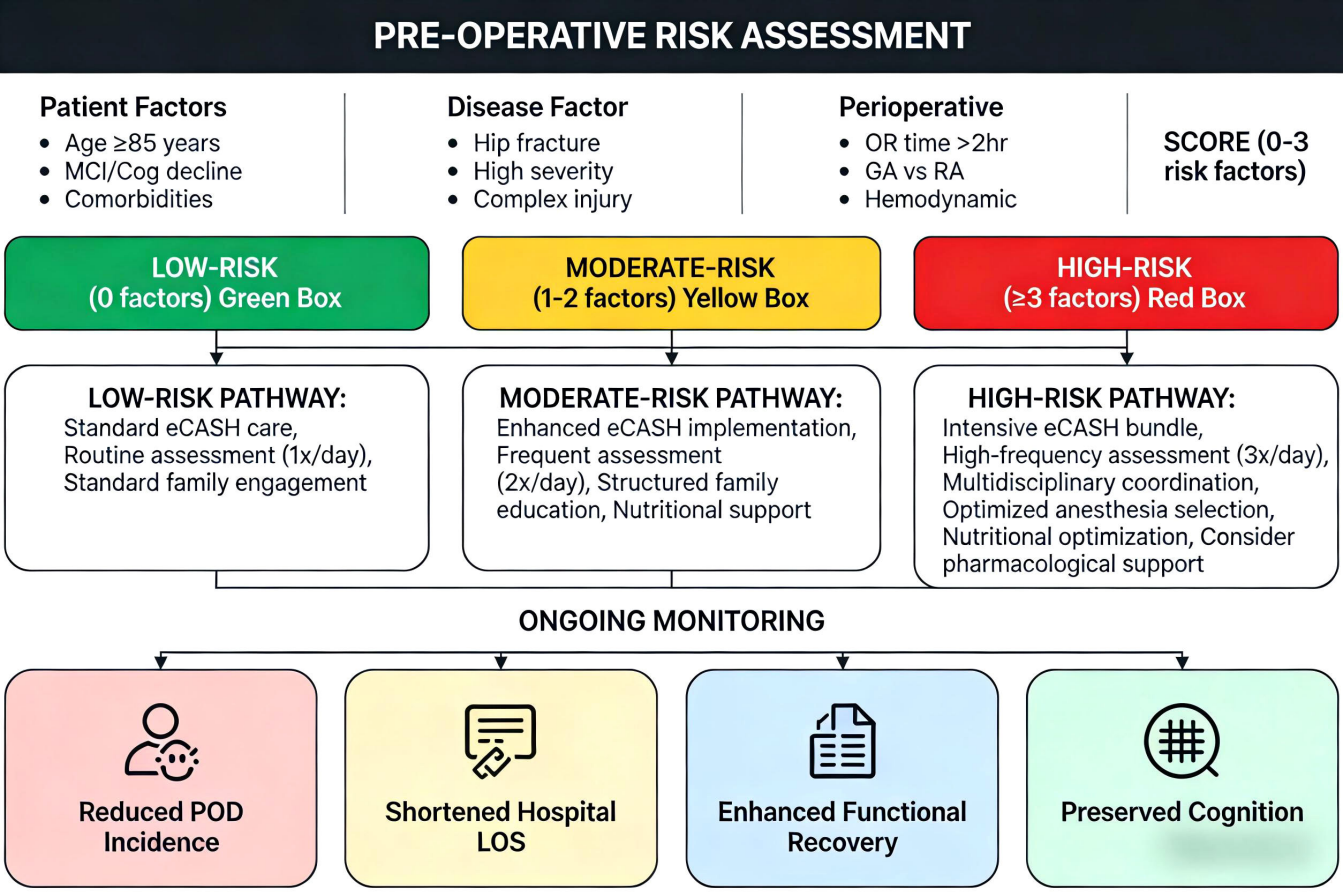
Risk stratification model and individualized algorithm.

Contemporary practice should incorporate formal risk assessment at preoperative evaluation, stratifying patients into risk tiers (low, moderate, high) and calibrating intervention intensity accordingly. This approach enables resource-appropriate prevention, allocating intensive interventions to highest-risk patients while providing evidence-based standard care to lower-risk individuals (**Figure 1**).

## 5 From evidence to practice: the eCASH bundle

**Figure 2:**
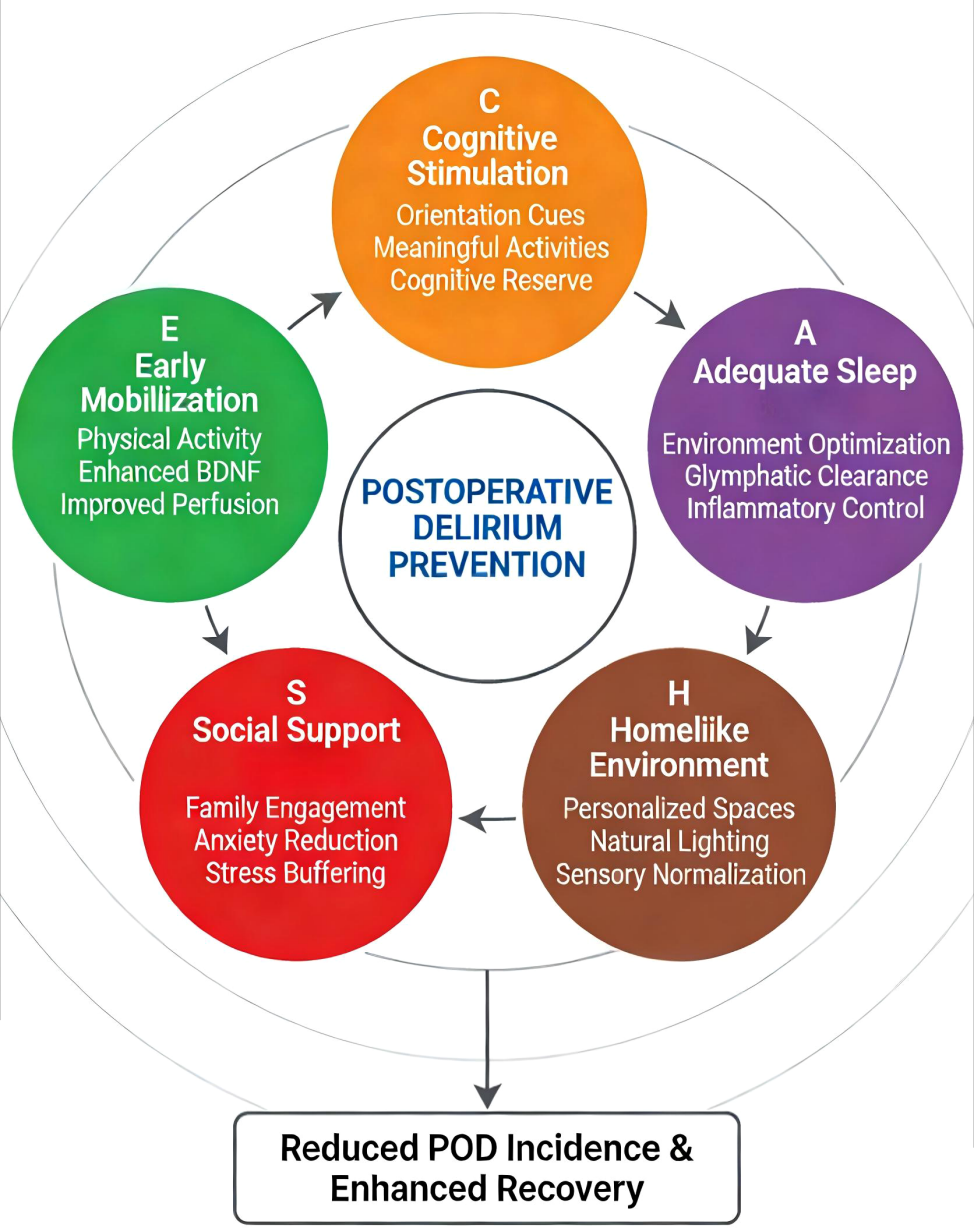
The eCASH bundle and mechanistic pathways.

The eCASH model—borrowed from intensive care delirium work and adapted to surgical wards—offers a memorable, evidence-based framework for non-drug interventions. Each component hits specific mechanisms while remaining doable at the bedside. Network meta-analyses have confirmed that multicomponent non-pharmacological interventions are among the most effective strategies for POD prevention (11) (**Figure 2**).

### 5.1 Early mobilization: more than just preventing deconditioning

Movement does more than keep muscles from wasting. Physical activity boosts brain-derived neurotrophic factor (BDNF), enhances neuroplasticity, and improves cerebral blood flow (12). One randomized trial found that combining respiratory training with therapeutic exercise, compared to routine care, significantly cut rates of acute postoperative cognitive dysfunction (12).

But “early mobilization” has to be individualized. For a patient fresh out of surgery, passive range-of-motion or even just turning in bed has neurobiological value. The core principle is movement itself—not any single protocol.

### 5.2 Cognitive stimulation: activating reserve

A cognitively intact person stuck in a sterile hospital room—white walls, no clock, no calendar, no familiar faces—can become disoriented just from sensory deprivation. Systematic cognitive stimulation works: orientation cues (clocks, calendars), structured conversation, family involvement, purposeful activities (13). Some hospitals have tried gamification and music therapy with promising early results. The key is adaptability.

### 5.3 Sleep optimization: clearing the brain’s waste

Hospital sleep is a disaster—frequent vital checks, beeping monitors, lights on and off at all hours. That wrecked sleep directly impairs the glymphatic system, which normally clears interstitial waste and inflammatory cytokines (4). Simple environmental changes—reducing noise, controlling light, minimizing unnecessary nighttime disruptions—demonstrably improve sleep and reduce delirium.

One unexpected finding: traditional Chinese medicine practices like auricular acupressure (ear point stimulation) have shown real benefits for sleep and delirium in some trials (1). That’s a nice reminder that evidence-based traditional approaches can enrich modern nursing care.

### 5.4 Social support and family engagement

Letting families visit freely and actively participate—helping with daily activities, supporting mobility, having real conversations—reduces anxiety, blunts stress responses, and keeps patients cognitively engaged (4). Familiar faces act as a neurobiological anchor against the fear and disorientation of acute illness. Family members become co-therapists, not visitors.

### 5.5 Homelike environment curation

Making a hospital room feel more like home—personal items, natural light, familiar objects—helps orient patients and reduce delirium through sensory normalization. This costs almost nothing but can have a real impact.

### 5.6 Synergistic effects

Here’s the key insight: these components don’t just add up—they multiply each other’s effects. Patients who get early mobilization plus good sleep plus family support plus cognitive stimulation show risk reductions far beyond what you’d predict by adding the individual benefits (5, 14). That’s why a full bundle beats picking and choosing.

## 6 Multidisciplinary team-based implementation: breaking down silos

No single discipline can prevent POD alone. Yet in many hospitals, surgeons, anesthesiologists, nurses, and rehab therapists operate in their own silos. Emerging evidence says that has to change.

One landmark trial compared routine care to a structured protocol delivered by a coordinated team—orthopedic surgeons, anesthesiologists, nurses, nutritionists, and physical therapists. The multidisciplinary approach cut POD incidence by about 40% (1). That huge effect probably comes from both the synergistic benefits of the interventions and the systems-level optimization of communication and care continuity. Major professional societies now explicitly recommend multidisciplinary collaboration as a core strategy to reduce perioperative neurocognitive disorders (15).

### 6.1 Essential building blocks

Successful multidisciplinary coordination requires a few non-negotiables:

Shared understanding – every team member knows what POD is and why it matters

Unified assessment tools – everyone uses CAM or Nu-DESC the same way

Explicit documentation – risk factors, monitoring results, and interventions are clearly recorded

Regular team briefings – identify high-risk patients who need extra attention

Clear escalation pathways – when cognitive decline is spotted, someone specific responds

### 6.2 Health information systems

Electronic health records can help. Specialized delirium modules enable systematic tracking, flag protocol deviations, and trigger timely responses. Automated alerts and standing orders for daily assessment turn quality improvement from a vague idea into a daily habit.

## 7 Real-world implementation: navigating barriers

Getting from a beautiful evidence base to what happens at 2 AM on a busy ward is never easy. Here are the real obstacles—and what actually works.

### 7.1 Primary barriers

Resource constraints. Bundled delirium prevention—especially early mobilization and frequent cognitive checks—requires adequate nurse staffing. Many surgical units run on ratios that make rigorous screening impossible (16). Not surprisingly, daytime versus night-shift prevention compliance tracks directly with staffing levels.

Knowledge gaps and cognitive biases. Some clinicians still believe POD is just an inevitable part of getting old or a “normal” post-op phase. That belief kills motivation. Systematic education showing that POD is preventable—and that prevention saves money and suffering—is essential.

Organizational inertia. New protocols demand leadership commitment, staff training, and workflow redesign. On already stretched surgical services, that’s a heavy lift.

### 7.2 Evidence-based solutions that actually work

Structured multilevel education – basic pathophysiology for everyone, diagnostic skills for nurses, intervention competencies for all team members.

Protocol standardization – simple, resource-appropriate protocols that specify how often to assess, who gets flagged, and what to do. Paper-based or electronic checklists reduce cognitive load.

Stakeholder engagement – involve senior clinicians, frontline staff, and patient/family reps from the start. Buy-in beats top-down mandates.

Rapid-cycle quality improvement – Plan-Do-Study-Act loops let you adapt to local barriers as you go. This is especially valuable for fitting evidence to your specific context.

### 7.3 eCASH in low-resource settings

Let’s be honest: not every hospital has the staff or budget for a full eCASH program with dedicated delirium nurses. But that doesn’t mean the framework is useless. Here’s how to adapt each component when resources are tight:

Early mobilization: Even a nursing assistant or family member can help with bedside range-of-motion or sitting up in a chair. No specialized equipment needed.

Cognitive stimulation: A whiteboard with the date, a calendar, and five minutes of orientation conversation at each shift costs nothing.

Sleep optimization: Earplugs and eye masks are cheap. Cluster nighttime care to reduce interruptions. Dim the lights after 9 PM.

Social support: Liberalize family visiting hours—this costs zero dollars. If families can’t be present, a tablet for video calls works wonders.

Homelike environment: Bring in a few personal photos from home. That’s free.

The principle is to do something rather than nothing, and to layer on more intensive elements as resources allow. A partial eCASH bundle is better than no bundle.

## 8 Risk-stratified individualization: beyond one-size-fits-all

The eCASH framework works for everyone, but intensity should vary by risk.

### 8.1 Matching intensity to risk

Take a low-risk 70-year-old with intact cognition having elective knee replacement. Basic early mobilization teaching, sleep hygiene advice, and standard family engagement are probably enough.

Now consider an 85-year-old with mild cognitive impairment, poor nutrition, and a history of stroke, coming in for emergency femoral fracture repair. That patient needs:

Intensive preoperative nutritional support

Neuraxial rather than general anesthesia if feasible

At least once-per-shift cognitive assessments

The full multidisciplinary team engaged

Consideration of low-dose atypical antipsychotics if behavioral symptoms break through non-drug interventions

### 8.2 Emerging AI applications

Machine learning models are getting better at predicting POD by integrating multiple clinical parameters (17). Eventually, we may get to “precision delirium medicine”—interventions targeted to specific molecular or physiological abnormalities rather than broad risk categories. That’s still in the future, but it’s coming.

## 9 Systematic assessment and monitoring: tools that work

Reliable recognition depends on using validated tools at regular intervals—not just when something seems wrong.

### 9.1 Recommended assessment instruments

The Nu-DESC scale (Nursing Delirium Screening Scale) is a practical bedside tool with just five items: disorientation, inappropriate behavior, inappropriate communication, hallucination/delusion, and psychomotor retardation. Each scores 0–2, total 0–10. A cutoff of ≥2 suggests possible delirium (7). Staff need brief training and scheduled assessment times—ideally twice daily for high-risk patients.

A practical note: delirium often worsens in early evening (“sundowning”). That’s a good time to pay extra attention.

### 9.2 Integration into routine practice

Electronic health records can automate alerts, generate standing orders for daily assessment, and publicly report unit-level POD rates. That kind of systematic approach builds accountability and sustains adherence over time.

## 10 Biomarkers and precision approaches: the emerging frontier

Clinical observation remains the gold standard, but biomarkers might eventually give us earlier, more objective detection.

### 10.1 Promising candidates

Cerebrospinal fluid and blood markers of neuroinflammation (IL-6, IL-1β, TNF-α), neuronal injury (tau, p-tau, neurofilament light chain), and glymphatic dysfunction all show promise in research (17). Neuroimaging markers—disrupted functional connectivity, altered blood-brain barrier permeability—may one day guide precision interventions.

### 10.2 Translational hurdles

But we’re not there yet. Bringing biomarkers into routine care requires prospective validation, cost-effectiveness data, and integration into bedside algorithms. The next decade of POD research will focus on closing that biomarker-to-bedside gap.

### 10.3 Newer non-pharmacological approaches

Two recent developments deserve mention as emerging non-pharmacological strategies. First, a double-blind randomized controlled trial by Gao et al. (2026) demonstrated that preoperative high-frequency (10 Hz) repetitive transcranial magnetic stimulation (rTMS) targeting the left dorsolateral prefrontal cortex (DLPFC) significantly reduced POD incidence in elderly patients undergoing non-cardiac major surgery (active rTMS: 8.1% vs. sham: 28.8%; RR, 0.22; 95% CI 0.10 to 0.46; *p* < 0.001) (18). This effect was accompanied by improvements in early postoperative pain, sleep quality, and anxiety scores, suggesting that rTMS may enhance cognitive reserve via modulation of prefrontal-limbic networks and anti-inflammatory pathways. Earlier work by Zhou et al. (2025) confirmed similar benefits in abdominal surgery populations (19), and existing evidence-based guidelines support the safety and therapeutic potential of rTMS for cognitive indications (20, 21).

Second, an ongoing multicenter randomized controlled trial (protocol published in 2026) is investigating whether electroacupuncture (EA) preconditioning at acupoints Zusanli (ST36) and Baihui (GV20) can reduce POD in elderly patients undergoing laparoscopic radical prostatectomy (22). The trial will enroll 212 participants and measure POD incidence using the 3D-CAM, alongside biomarkers such as plasma P-tau 217 and S100β. This research builds on evidence that EA may attenuate neuroinflammation and preserve cognitive function (22). If confirmed, EA would offer a safe, culturally adaptable non-pharmacological strategy, particularly valuable in settings where advanced technologies like rTMS are unavailable.

## 11 Beyond acute hospitalization: discharge and continuity

POD doesn’t always end when the patient leaves the hospital. Some patients have persistent cognitive difficulties that need follow-up.

### 11.1 Discharge planning

At discharge, do a formal cognitive assessment. Document any residual deficits and determine rehabilitation needs. Then educate the family: what to expect in terms of activity progression, cognitive recovery, and warning signs of delayed improvement.

### 11.2 Post-discharge monitoring

Telehealth follow-up—by phone or video—can track cognitive recovery and catch delayed complications. As technology improves, remote assessment becomes more feasible. And community rehab providers need explicit handoffs about the patient’s POD history and cognitive vulnerabilities. Continuity matters.

## 12 Health economic dimensions: cost-effectiveness of prevention

Prevention programs cost money upfront: staff training, assessment tools, intervention time. But the evidence increasingly says they save money overall.

One comprehensive analysis compared systematic delirium risk prediction plus non-pharmacological prevention to routine care (14). The prevention program had higher initial costs but generated net savings when you counted the full cycle: shorter hospital stays, fewer transfers to post-acute care, lower complication rates, and better functional outcomes.

Other economic evaluations have reached similar conclusions. A systematic review of delirium prevention in older hospitalized patients found that multi-component programs consistently reduced hospital length of stay and overall costs, even after accounting for implementation expenses. The savings come from avoided complications—falls, pressure injuries, functional decline, institutionalization—that are common after a POD episode.

For hospital administrators and resource allocation committees, the economic case is increasingly solid. Prevention isn’t a cost—it’s an investment with a positive return. In resource-limited settings, that argument becomes even more powerful: doing nothing is actually more expensive in the long run.

## 13 Discussion: negative and controversial results

No review is complete without acknowledging where the evidence is messy, contradictory, or just plain negative.

First, not all studies show a benefit from multi-component delirium prevention. Some well-designed trials have reported neutral results, especially those implemented in highly resource-constrained settings or where fidelity to the protocol was low. This suggests that how you implement may matter as much as what you implement. A recent network meta-analysis of non-pharmacological interventions noted substantial heterogeneity in effect sizes across studies, highlighting the importance of context and intervention fidelity (11).

Second, the role of antipsychotics for prevention remains controversial. While haloperidol and atypical antipsychotics are sometimes used off-label, randomized trials have largely failed to show benefit for prevention, and they carry significant risks (QT prolongation, extrapyramidal symptoms, increased mortality in dementia patients). Current guidelines recommend reserving them for short-term management of severe agitation that fails non-drug measures, not routine prophylaxis (10).

Third, the evidence for specific eCASH components varies in quality. Early mobilization and sleep optimization have strong support; the independent contribution of “homelike environment” is harder to isolate and may be more context-dependent.

Fourth, most studies exclude patients with severe dementia or delirium at baseline, so the evidence may not generalize to the sickest patients. And publication bias—toward positive results—is always a concern in this field.

Acknowledging these limitations doesn’t undermine the overall message; it strengthens it by showing we’ve thought critically about the evidence.

## 14 Conclusions and recommendations

POD in elderly orthopedic patients is not inevitable. We now have robust evidence-based strategies to prevent it. The core mechanisms—neuroinflammation, neurotransmitter imbalance, glymphatic dysfunction—explain why aging brains are vulnerable and point toward rational interventions.

### 14.1 An integrated approach that works

The following package offers the best chance of meaningful POD reduction:

Systematic risk stratification at preoperative evaluation, using validated tools to calibrate intervention intensity

eCASH-informed bundled care – early mobilization, cognitive stimulation, sleep optimization, family involvement, environmental normalization

Multidisciplinary team coordination – surgery, anesthesia, nursing, rehabilitation, and geriatrics when available

Standardized assessment protocols – validated tools (CAM/Nu-DESC) at specified intervals, with results communicated across shifts

Quality improvement methodology – rapid-cycle testing, stakeholder engagement, local adaptation

Staff education and competency – everyone understands POD pathophysiology, recognizes early signs, and can deliver interventions

Continuity of care – through discharge and into post-acute follow-up

### 14.2 Future directions

Biomarkers, AI-assisted risk prediction, and remote monitoring will refine prevention further. But the basics—clinical recognition, evidence-based action, sustained multidisciplinary commitment—are already within reach of any surgical program, regardless of resource level.

### 14.3 Final thoughts

For surgical teams that truly want to improve outcomes for older adults, the case for systematic POD prevention is now overwhelming. Implementation doesn’t start with a new machine or a billion-dollar budget. It starts with an intellectual commitment: acknowledging that delirium is preventable, setting clear institutional priorities, and mobilizing the people and resources you already have toward this urgent clinical challenge. The evidence is ready. The question is whether we are.
